# Identification of lipoxygenase (*LOX*) genes from legumes and their responses in wild type and cultivated peanut upon *Aspergillus flavus* infection

**DOI:** 10.1038/srep35245

**Published:** 2016-10-12

**Authors:** Hui Song, Pengfei Wang, Changsheng Li, Suoyi Han, Javier Lopez-Baltazar, Xinyou Zhang, Xingjun Wang

**Affiliations:** 1 Biotechnology Research Center, Shandong Academy of Agricultural Sciences; Shandong Provincial Key laboratory of Crop Genetic Improvement, Ecology and Physiology, Jinan 250100, PR China; 2 Henan Academy of Agricultural Sciences, Zhengzhou 450002, PR China; 3 Departamento de Ingenierías, Instituto Tecnológico del Valle de Oaxaca, Oaxaca, Mexico

## Abstract

Lipoxygenase (*LOX*) genes are widely distributed in plants and play crucial roles in resistance to biotic and abiotic stress. Although they have been characterized in various plants, little is known about the evolution of legume *LOX* genes. In this study, we identified 122 full-length *LOX* genes in *Arachis duranensis*, *Arachis ipaënsis*, *Cajanus cajan*, *Cicer arietinum*, *Glycine max*, *Lotus japonicus* and *Medicago truncatula*. In total, 64 orthologous and 36 paralogous genes were identified. The full-length, polycystin-1, lipoxygenase, alpha-toxin (PLAT) and lipoxygenase domain sequences from orthologous and paralogous genes exhibited a signature of purifying selection. However, purifying selection influenced orthologues more than paralogues, indicating greater functional conservation of orthologues than paralogues. Neutrality and effective number of codons plot results showed that natural selection primarily shapes codon usage, except for *C. arietinum*, *L. japonicas* and *M. truncatula LOX* genes. GCG, ACG, UCG, CGG and CCG codons exhibited low relative synonymous codon usage (RSCU) values, while CCA, GGA, GCU, CUU and GUU had high RSCU values, indicating that the latter codons are strongly preferred. *LOX* expression patterns differed significantly between wild-type peanut and cultivated peanut infected with *Aspergillus flavus*, which could explain the divergent disease resistance of wild progenitor and cultivars.

Lipoxygenases (LOXs, EC1.13.11.12) are non-heme iron-containing enzymes that are widely distributed in plants and animals. LOX proteins catalyse the oxidation of polyunsaturated fatty acids into unsaturated fatty acid hydroperoxides^1^. LOX can induce synthesis of oxylipins, including acyclic and cyclic compounds^2^. Oxylipins can activate diverse pathways, including those associated with hydroperoxide lyase (HPL), peroxygenase (POX), allene oxide synthase (AOS), and divinyl ether synthase (DES)^2^,^3^. LOX proteins contain two major domains, a polycystin-1, lipoxygenase, alpha-toxin (PLAT) domain at the N-terminus and a lipoxygenase domain at the C-terminus^4^. The PLAT domain, which contains an eight-stranded antiparallel β-barrel, is found in a variety of membrane- or lipid-associated proteins^5^. This domain can bind to procolipase, which mediates membrane associations, such as polycystin-1 function^5^,^6^. The plant lipoxygenase domain contains about 38 amino acids with five conserved histidines, which are involved in iron binding^7^. LOX proteins can be classified into two types, 9-LOX and 13-LOX, based on their specificity of fatty acid oxygenation of linoleic acids (LA, 18:2) and linolenic acids (LeA, 18:3)^8^. Moreover, 13-LOX proteins can be further classified into two subgroups, type I 13-LOX and type II 13-LOX. Type I 13-LOX proteins harbour no transit peptides, exhibit high sequence similarity (>75%) between one another and are usually cytosolically localized^9^. Type II 13-LOX proteins are chloroplastic proteins carrying N-terminal transit peptides and showing moderate sequence similarity (>35%) to one another^9^.

To date, many *LOX* genes have been identified or cloned from plants including *Arabidopsis thaliana*^10^, *Glycine max*^11^, *Medicago truncatula*^11^, *Pyrus bretschneideri*^12^ and *Vitis vinifera*^2^ One review has reported that plant *LOX* genes are involved in responses to many pathways such as growth and developmental processes and resistance to abiotic stress^2^. Zhang, *et al*.^13^ showed that the expression levels of 18 *Cucumis melo LOX* genes are differentially regulated during melon development and ripening. In apple, *MdLOX1a* and *MdLOX5e* are involved in fruit aroma volatile production^14^. Yang, *et al*.^15^ found that 12 cucumber *LOX* genes are involved in response to abiotic stresses, including cold (4 °C), NaCl (200 mM) and KCl (200 mM). In *Panax ginseng*, *PgLOX3* expression is increased under water deficit stress^16^. LOX can catalysed the initial step of the methyl jasmonate (JA) pathway, but little is known about its regulatory mechanism^2^,^17^. Chen, *et al*.^4^ found that 20 poplar *LOX* genes are regulated by methyl jasmonate (MeJA) treatment. *LOX* transcript levels in maize seeds were positively associated with resistance to fungi^18^,^19^. Podolyan, *et al*.^3^ reported that the expression of *VvLOXC* and *VvLOXO* increased under mechanical wounding and *Botrytis cinerea* infection in *V. vinifera*, while *VvLOXA* expression decreased in berries after pathogen infection. In cultivated peanut (*Arachis hypogaea*), transcriptome data showed at least 19 *LOX* genes were differentially expressed after *Aspergillus flavus* infection^20^,^21^.

Recently, the genome sequences of *Arachis duranensis*, *Arachis ipaënsis*, *Cajanus cajan*, *Cicer arietinum*, *Glycine max*, *Lotus japonicus* and *Medicago truncatula* have been completed and released^22^,^23^,^24^,^25^,^26^,^27^,^28^. These species belong to the Papilionoideae subfamily, and their phylogenetic relationships are shown in Fig. 1. The availability of whole genome sequences enables the comparative analysis of *LOX* genes in these legume species. Previous studies have identified 143 unique *LOX* genes from four model and seventeen crop plants, including monocotyledonous and dicotyledonous plants, which can be classified into two subfamilies based on a predicted chloroplast-targeting peptide^29^. However, these sequences were collected from public databases such as NCBI and UniProt in previous study^29^. In this study, we identified *LOX* genes from the seven aforementioned leguminous genomes using a bioinformatics approach. Both the overall phylogenetic relationships and codon usage patterns were analyzed. The expression patterns of some *LOX* genes in *A. duranensis* and its orthologous genes in cultivated peanut (*A. hypogeae* L.) were determined after *A. flavus* infection using quantitative real-time PCR (qRT-PCR). This provides helpful insights into the evolution of *LOX* genes and their function in *A. flavus* resistance.

## Results and discussion

### Identification and classification of *LOX* genes in seven legumes

We identified *LOX* genes in *A. duranensis* (*AdLOX*), *A. ipaënsis* (*AiLOX*), *C. arietinum* (*CaLOX*), *C. cajan* (*CcLOX*), *G. max* (*GmLOX*), *L. japonicus* (*LjLOX*) and *M. truncatula* (*MtLOX*) using a bioinformatics approach. From these genome sequences, we retained 122 full-length *LOX* gene sequences for phylogenetic analysis, including 14 *AdLOX*, 13 *AiLOX*, 10 *CaLOX*, 16 *CcLOX*, 36 *GmLOX*, 5 *LjLOX* and 28 *MtLOX* sequences (Table 1 and Table S1). A total of 19 *GmLOX* and 15 *MtLOX* sequences have been released in a previous study that used four *G. max* and two *M. truncatula* regions^11^. However, in this study, we identified 34 *GmLOX* and 28 *MtLOX* sequences to identify additional *LOX* sequences. The number of *LOX* genes identified was significantly positively correlated with the number of whole-genome duplications (WGDs, *r* = 0.81, *P* < 0.05), but had no correlation with genome size (r = 0.09, *P* > 0.05), indicating WGD events directly affect the number of *LOX* genes. Fox example, *G. max* (1100 Mb, 3 WGD) contains 36 *LOX* genes, and *A. ipaënsis* (1560 Mb, 1 WGD) contains 13 *LOX* genes, while *M. truncatula* (375 Mb, 3 WGD) contains 28 *LOX* genes (Table 1). Shin, *et al*.^11^ found that *GmLOX* and *MtLOX* genes have a common ancestor, but *GmLOX* genes expanded through an ancient polyploidy event prior to taxon divergence, followed by a soybean-specific duplication. Further, there is positive but not significant correlation (*r* = 0.92, *P* > 0.05) between the number of *LOX* genes and genome size among species exhibiting an equal number of WGD events. One WGD event each was detected in *A. duranensis* (1250 Mb), *A. ipaënsis* (1560 Mb), *C. arietinum* (738 Mb) and *L. japonicas* (315 Mb) (Table 1). Accordingly, the numbers of *LOX* genes are 14 (*AdLOX*), 13 (*AiLOX*), 10 (*CaLOX*) and 5 (*LjLOX*), respectively (Table 1).

**Figure 2:**
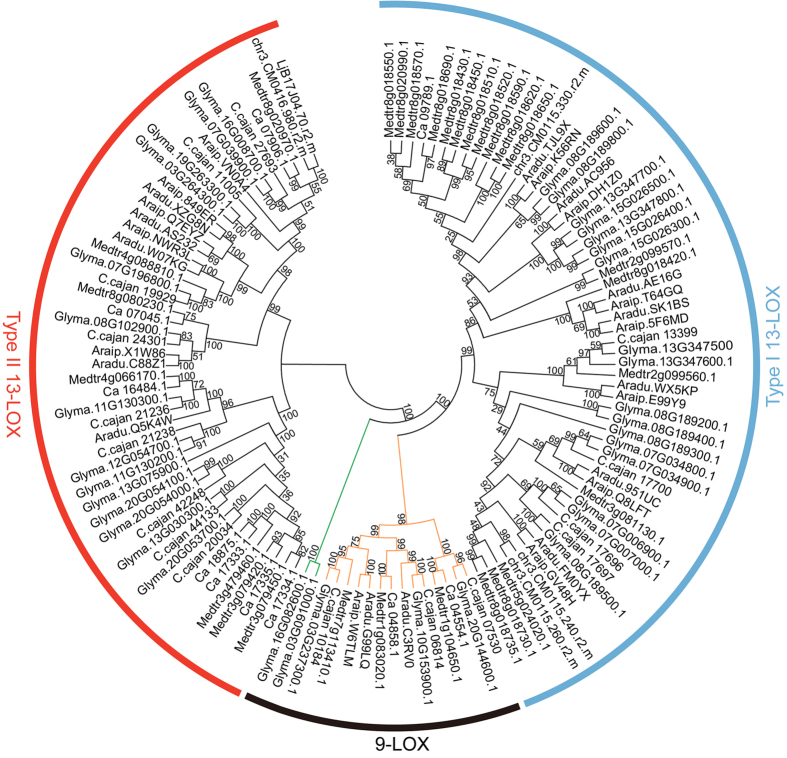
Phylogenetic tree of LOX amino acid sequences in seven legumes. The phylogenetic tree was constructed using MEGA 6.0. The phylogenetic tree was estimated using maximum likelihood with the Jones-Taylor-Thornton (JTT) model and branch support estimates are based on 1,000 bootstrap replicates.

A phylogenetic tree was generated using amino acid sequences, and it shows three clear clades, including 9-LOX, type I 13-LOX and type II 13-LOX (Fig. 2). Compared to 13-LOX, relative fewer 9-LOX amino acid sequences were identified. In 9-LOX, only one paralogous gene pair (Glyma.03G091000.1 and Glyma.16G082600.1) was identified (Fig. 2, green colour), suggesting few duplication events occurred in 9-LOX. Most gene families can be classified into several groups. Fox example, WRKY proteins can be classified into three groups, and group II can be further classified into five subgroups based on the numbers and types of domains^30^,^31^,^32^. Nucleotide-binding site (NBS)-Leucine-rich repeat (LRR) proteins can be further classified into TIR-NBS-LRR and CNL-NBS-LRR based on the presence of toll/mammalian interleukin-1 receptor (TIR) or coiled-coil (CC) domains at N-terminuses^33^,^34^,^35^. The origin of these proteins is always a key research focus^35^,^36^. Little is known about the origin of LOX proteins. Typically, researchers have classified LOX proteins according to fatty acid oxygenation specificity^8^. Based on a phylogenetic tree generated in this study, the divergence of 9-LOX occurred between the divergence of type I 13-LOX and type II 13-LOX (Fig. 2). Furthermore, 9-LOX sequences were clustered into two groups (Fig. 2, shown in green and orange), indicating that 9-LOX sequences have independent origins.

### *LOX* homologous genes in seven legumes

**Figure 3:**
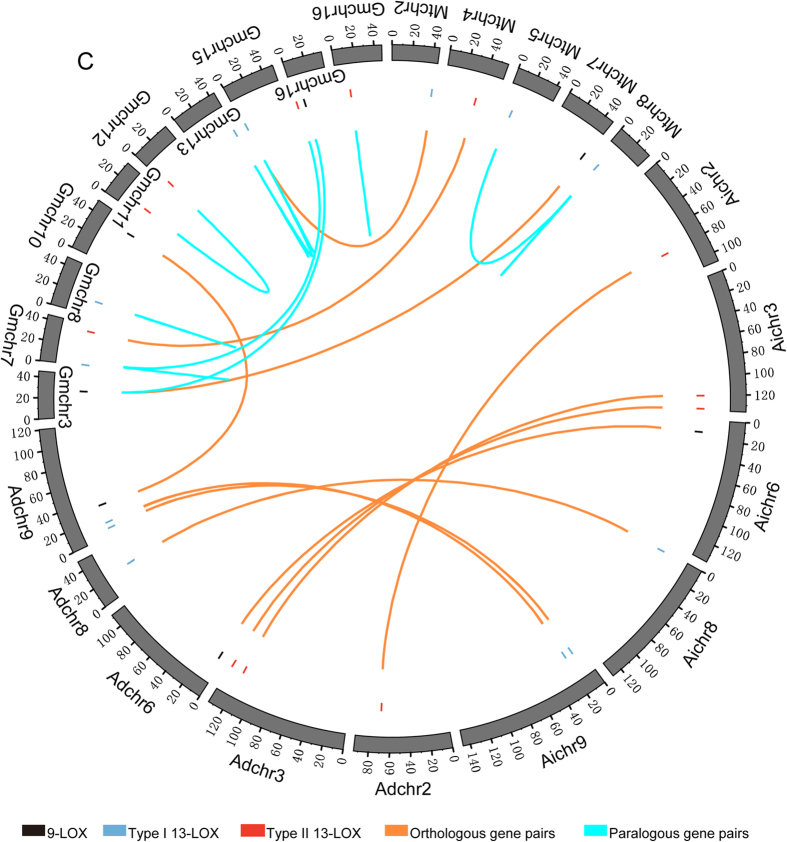
Chromosomal location and homologous gene relationships among *LOX* genes from *A.* *duranensis*, *A. ipaënsis*, *G. max* and *M. truncatula*. The chromosomal location information of *LOX* genes was obtained from the source websites for each sequence. The map was generated using Circos v0.69.

**Figure 4:**
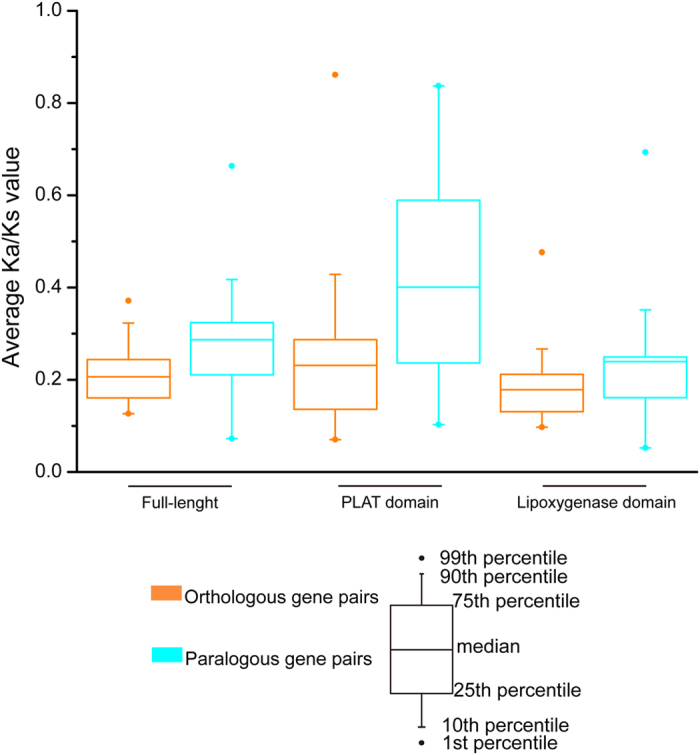
*K*_a_/*K*_s_ values calculated using full-length, PLAT and lipoxygenase domains from homologous genes. PAL2NAL was used to convert amino acid sequences into the corresponding nucleotide sequences. PAML 4.0 was used to calculate the nonsynonymous/synonymous substitution (*K*_a_/*K*_s_) ratio. *K*_a_/*K*_s_ values of 1, >1 and <1 indicated neutral, positive, and purifying selection, respectively.

In this study, 63 orthologous and 36 paralogous genes were identified (Fig. 3). To determine if selection pressures were uniform among orthologues and paralogues in full-length *LOX* genes as well as PLAT and lipoxygenase domain sequences, we calculated the nonsynonymous/synonymous (*K*_a_/*K*_s_) values of these three types of sequence. All estimated *K*_a_/*K*_s_ values were less than 1 (Fig. S1), suggesting each of these sequences underwent purifying selection. However, *K*_a_/*K*_s_ values of paralogous genes exceeded those of orthologous genes among full-length *LOX* genes as well as PLAT and lipoxygenase domain sequences (*t*-test, *P* < 0.05, Fig. 4). The results suggested constrained purifying selection influenced orthologues more than paralogues, indicating that the biological function of orthologues is more conserved than that of paralogues. In addition, purifying selection on the lipoxygenase domain has exceeded that on the PLAT domain among orthologous and paralogous genes, indicating the biological function of the lipoxygenase domain is more conserved than that of PLAT domains.

*CcLOX*, *CaLOX* and *LjLOX* genes were excluded from the chromosomal location analysis because most of these genes lacked localization information. *GmLOX* genes were distributed across 11 of 20 chromosomes, *MtLOX* genes were detected in 7 of 8 chromosomes and *AdLOX* and *AiLOX* genes were identified on 6 of 10 chromosomes, respectively (Fig. 3, Table S1). Many paralogous genes were among *GmLOX* and *MtLOX* sequences, while no paralogous genes were found among *AdLOX* and *AiLOX* sequences (Fig. 3). However, most orthologous genes were identified among *AdLOX* and *AiLOX* sequences in corresponding chromosomes (Fig. 3), suggesting that the biological functions of orthologous genes among *AdLOX* and *AiLOX* genes were more conserved than those of *GmLOX* and *MtLOX* genes.

*A. duranensis* and *A. ipaënsis* diverged 2.9–3.5 million years ago (MYA)^37^, and the cultivated peanut was domesticated 3500–4500 years ago^24^. Full genome sequences revealed most orthologous genes appeared in the *A. duranensis* and *A. ipaënsis* genomes^24^. However, it is difficult to distinguish homologous genes from *A. duranensis* and *A. ipaënsis* in the cultivated peanut genome. In this study, we found that most *AiLOX* genes were longer than their homologous *AdLOX* genes (Fig. S2). The difference between homologous *AdLOX* and *AiLOX* genes is attributed to intron length differences, especially the first intron. This result can be helpful for the future analysis and assembly of the cultivated peanut genome.

### Codon usage bias of *LOX* genes in seven legumes

The average GC content was lower than the AT content in the seven legume species (Table 2). We found that the GC content at the three codon positions was, in decreasing order, GC1 (GC at the first codon position) > GC3 (GC at the third codon position) > GC2 (GC at the second codon position) in *AdLOX*, *AiLOX*, *CcLOX*, *GmLOX* and *LjLOX*. However, in *CaLOX* and *MtLOX*, GC content was as follows: GC1 > GC2 > GC3 (Table 2). In a neutrality plot, if the correlation between GC12 (average of GC1 and GC2) and GC3 is significant and the slope of the regression is close to 1, mutation pressure is the main force shaping codon usage^38^. When natural selection is the dominant factor, the slope of the regression is close to 0^38^. In this study, GC12 and GC3 from *AdLOX* (*r* = 0.76393, *P* < 0.05 and slope = 0.1786), *AiLOX* (*r* = 0.77378, *P* < 0.05 and slope = 0.21234), *CcLOX* (*r* = 0.82218, *P* < 0.05 and slope = 0.16592) and *GmLOX* (*r* = 0.49242, *P* < 0.05 and slope = 0.1393) showed a significant positive correlation, and the slope was close to 0 (Fig. S3). These results suggested that codon usage was primarily shaped by natural selection. GC12 and GC3 from *CaLOX*, *LjLOX* and *MtLOX* showed a positive correlation but it was not significant (Fig. S3). These results indicated that different evolutionary pressures shaped variation in these legumes. If codons are constrained by mutation pressure (i.e. nucleotide composition), the gene would be on the curve in an effective number of codons (ENC) plot^39^. Genes from seven legumes were all below the curve line, suggesting that other evolutionary pressures (i.e. natural selection) likely influence codon usage (Fig. S4). Nevertheless, if GC3s values (GC content at synonymous codons) are narrow, natural selection is involved in codon usage patterns^40^. In the seven legumes, GC3s values were narrowly distributed (*AdLOX*: 0.325–0.434, *AiLOX*: 0.314–0.424, *CaLOX*: 0.267–0.339, *CcLOX*: 0.303–0.557, *GmLOX*: 0.312–0.522, *LjLOX*: 0.385–0.449, and *MtLOX*: 0.276–0.362, Fig. S4). Neutrality and ENC plots showed that natural selection is main force shaping codon usage, while the codon usage of *CaLOX*, *LjLOX* and *MtLOX* may involve other processes.

**Figure 5:**
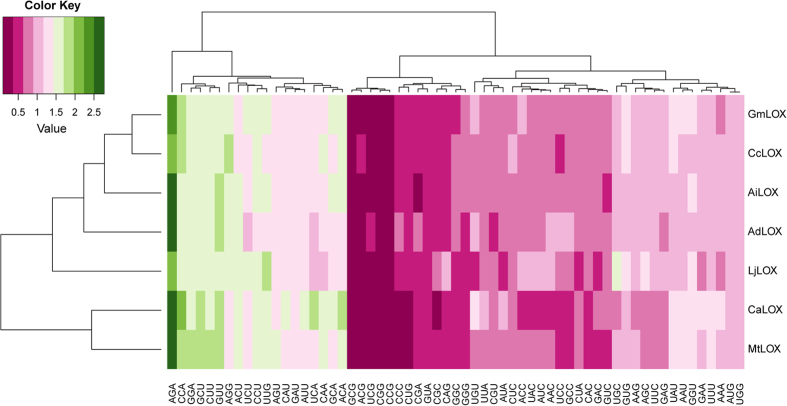
Hierarchal clustering of RSCU values for each codon in seven legumes. Relative synonymous codon usage (RSCU) was calculated using codonW. RSCU < 1 indicated the codons are less used, and RSCU > 1 indicated that the codons are used more frequently than expected.

Relative synonymous codon usage (RSCU) is the observed frequency of a codon divided by the expected frequency. RSCU < 1 indicates less-used codons, and RSCU > 1 indicates that the codons are used more frequently than expected^41^. *LOX* genes in the seven legumes showed a strong preference for AT-ending codons based on the above criterion (RSCU > 1, Fig. 5 and Table S2). The higher AT contents at the third position than GC contents may explain these results (Table 2). Based on a heat map, RSCU values can be classified into two groups (Fig. 5). The low RSCU group included GCG, ACG, UCG, CGG and CCG. The RSCU value (~2.5) of AGA was the highest and CCA, GGA, GCU, CUU and GUU had relatively high RSCU values (~1.5); these were included in the high RSCU group. These results indicated GCG, ACG, UCG, CGG and CCG are not preferentially used codons and CCA, GGA, GCU, CUU and GUU are strongly preferred. Seven legume *LOX* genes could be classified into two groups based on RSCU values. *AdLOX*, *AiLOX*, *CcLOX*, *GmLOX* and *LjLOX* clustered into one group, and *CaLOX* and *MtLOX* were included in another group. These results are consistent with the GC content results. The GC contents at the three codon positions in *CaLOX* and *MtLOX* were similar and the five additional *LOX* genes had nearly equal GC contents at the three positions, suggesting GC content at the three positions may influence RSCU values.

### Expression of *LOX* genes in wild and cultivated peanuts after *A. flavus* infection

Previous studies indicated that wild peanut is more resistant to diseases than cultivated peanut, and transferring resistance genes from wild peanut to cultivars can improve disease resistance in cultivated peanut^42^. Recently, sequencing results indicated that the genome size of *A. hypogaea* (~2.8 Gb) is similar to the sum of the *A. duranensis* (~1.25 Gb) and *A. ipaënsis* genomes (~1.56 Gb), indicating that most genes from the two wild peanuts are probably present in cultivated peanut^24^. Here, we hypothesize that *LOX* gene activities explain the higher resistance in wild peanut than cultivated peanut.

**Figure 6:**
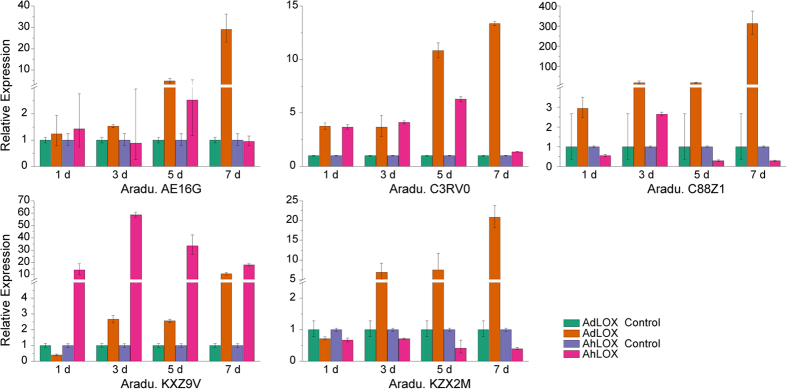
Expression of *LOX* genes in *A. duranensis* and *A. hypogaea* after *A.* *flavus* infection. The *Y*-axis indicates the relative expression level and the *X*-axis (1, 3, 5, and 7 d) indicates the number of days after *A. flavus* infection. The standard errors are shown using vertical lines.

*A*. *flavus* produces carcinogenic mycotoxins known as aflatoxins, which are toxic to animals, including humans. Aflatoxin contamination is an important factor limiting peanut production. According to a previous study, *LOX* gene expression is related to the response to *A*. *flavus* infection^20^. In this study, expression patterns of five *LOX* genes were compared between *A. duranensis* and *A. hypogaea* using qRT-PCR after *A. flavus* infection. The expression of *AE16G* (type I 13-LOX) steadily increased upon *A. flavus* infection. After 7 days of infection, the RNA level was more than 10 times higher than the level observed on the first day after infection in *A. duranensis* (Fig. 6). However, the expression of this gene did not change significantly in *A. hypogaea* after infection (Fig. 6). Similar expression patterns were found in *C88Z1* (type II 13-LOX) and *KZX2M* (9-LOX, partial sequence) (Fig. 6). Increased expression of *C3RV0* (9-LOX) was observed in both *A. duranensis* and *A. hypogaea* during the first 5 days of infection. On the seventh day after infection, the expression of *C3RV0* continued to increase in *A. duranensis*, but decreased drastically in *A. hypogaea* (Fig. 6). *KXZ9V* (type I 13-LOX, partial sequence) showed a different expression pattern than that of the other four *LOX* genes upon *A. flavus* infection (Fig. 6). Lower expression levels were observed in *A. duranensis* than in *A. hypogaea*. Expression reached the highest level on the third day after infection, and then decreased beginning on the fifth day (Fig. 6). It is noteworthy that the expression levels in *A. duranensis* continuously increased after *A. flavus* infection, but not in *A. hypogeae* (Fig. 6).

Orthologous genes in polyploids and their parents have at least three possible fates, including expression-level dominance, transgressive segregation and homeolog expression bias^43^. The results of this study showed that total gene expression in cultivated peanut is lower or higher than that in diploid parents, consistent with the transgressive segregation model. Many processes, such as DNA methylation and *cis*-regulation, may explain this phenomenon according to previous reviews^43^,^44^. DNA methylation on cytosines at CG, CHG and CHH sites is an important epigenetic factor influencing transcriptomic changes^44^. In *Arabidopsis*, DNA methylation levels differ between F1 hybrids and parent plants (*A. thaliana Ler* and *C24*); 18–26% of GC sites exhibit DNA methylation in parents compared with 36–37% in hybrids^45^. A total of 77 genes sensitive to methylome remodelling are transcriptionally repressed in F1 hybrids^45^. Lee and Chen^46^ demonstrated that DNA methylation regulation is involved in the expression of orthologous genes in *Arabidopsis suecica* and its diploid parents *A. thaliana* and *Cardaminopsis arenosa*. Liu, *et al*.^47^ found the expression level of *Gossypium barbadense WRKY1* is higher than that in *Gossypium hirsutum WRKY1* after *Verticillium dahliae* infection, and the *GhWRKY1* promoter lacks an ethylene-responsive element compared to the *GBWRKY1* promoter, indicating promoter differences probably resulted in differences in expression patterns. Understanding the expression regulation of disease-resistance genes in tetraploid peanut may facilitate the development of an efficient method to improve disease resistance in cultivated peanut.

## Conclusion

In this study, we identified *LOX* genes in seven legumes. The full-length, PLAT and lipoxygenase domain sequences from orthologous and paralogous genes exhibited signatures of purifying selection. Constrained purifying selection influenced orthologous genes more than paralogous genes. Natural selection was the driving force shaping codon usage, while *CaLOX*, *LjLOX* and *MtLOX* genes may have been influenced by other processes. Legume *LOX* genes preferentially used CCA, GGA, GCU, CUU and GUU. The expression pattern of *LOX* genes differed significantly between wild-type and cultivated peanuts.

## Materials and Methods

### *LOX* sequences in seven legume genomes

We downloaded the genome sequences of *A. duranensis* (http://peanutbase.org/), *A. ipaënsis* (http://peanutbase.org/), *C. cajan* (http://gigadb.org/dataset/100028), *C. arietinum* (http://nipgr.res.in/CGAP/home.php), *G. max* (https://phytozome.jgi.doe. gov/pz/portal.html), *L. japonicus* (http://www.kazusa. or.jp/lotus/) and *M. truncatula* (http://jcvi.org/medicago/display.php?pageName = General&section = Download). To obtain LOX coding sequences, we downloaded the HMM profile of the lipoxygenase domain (PF00305) from pfam database (http://pfam.janelia.org). Each *LOX* gene was extracted using the HMMER program^48^. To verify the reliability of the results, all amino acid sequences were checked in the pfam database. If PLAT (PF01477) and lipoxygenase domains were both present, the sequence was consider a *LOX* gene^4^.

### Phylogenetic analysis

Multiple sequence alignment and phylogenetic tree construction were carried out using MAFFT 7.0^49^ and MEGA 6.0^50^, respectively. The phylogenetic tree was estimated using maximum likelihood with the Jones-Taylor-Thornton (JTT) model based on 1,000 bootstrap replicates. If genes from different species clustered in the phylogenetic tree with a bootstrap value of greater than 70, these genes were considered orthologous. Similarly, if genes from a single species clustered in a phylogenetic tree with a bootstrap value of greater than 70, these genes were considered paralogous. The PAL2NAL program^51^ was used for the conversion of amino acid sequences into the corresponding nucleotide sequences. PAML 4.0^52^ was used to calculate the nonsynonymous/synonymous substitution (*K*_a_/*K*_s_) ratio. Generally, *K*_a_/*K*_s_ values of 1, >1 and <1 indicate neutral, positive, and purifying selection, respectively.

The chromosomal locations of *LOX* genes in legume plant genomes were obtained from the source website for each sequence. The map was generated using the Circos v0.69 program^53^.

GC content at synonymous codons (GC3s), effective number of codons (ENC) and relative synonymous codon usage (RSCU) were calculated using the codonW program (http://codonw.sourceforge.net). GC1, GC2 and GC3 values were calculated using an in-house Perl script.

### Gene expression analysis

The *A. flavus* inoculation method was described by Zhang, *et al*.^20^ Briefly, mature peanut seeds were surface-sterilized and placed on humid filter paper at 28 °C for 3 days. The germinated peanut seeds were used for inoculation by immersion in a suspension of ~3 × 10^7^ spores/ml of *A. flavus*. Seeds immersed in sterile distilled water were used as controls. Seeds were placed in Petri dishes at 28 °C, and were harvested at 1, 3, 5 and 7 days after treatment.

qRT-PCR primers were designed based on the *A. duranensis* genome sequence using the Beacon Designer 8.0 program. The primers can distinguish genes from the A subgenome and B subgenome in cultivated peanut (Luhua14). The primers are shown in Table S3.

The hexadecyltrimethylammonium bromide (CTAB) method was used to extract total RNA. qRT-PCR was carried out using Fast Start Universal SYBR Green Master (ROX) and a 7500 Real-time PCR machine (ABI).

## Additional Information

**How to cite this article**: Song, H. *et al*. Identification of lipoxygenase (*LOX*) genes from legumes and their responses in wild type and cultivated peanut upon *Aspergillus flavus* infection. *Sci. Rep.* **6**, 35245; doi: 10.1038/srep35245 (2016).

## Supplementary Material

Supplementary Information

## Figures and Tables

**Figure 1:**
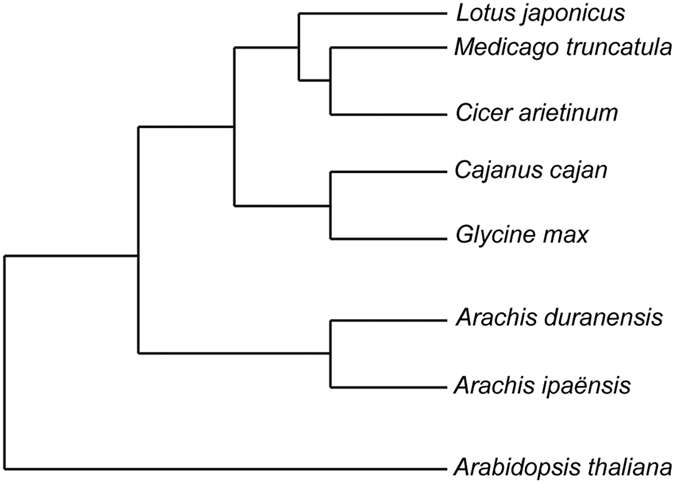
Phylogenetic tree of seven legumes. The topology of phylogenetic tree is revised based on references 22 and 23.

**Table 1:** The number and type of *LOX* genes in seven legumes.

Name	9-LOX	Type I 13-LOX	Type II 13-LOX	Total	Genome size	Whole-genome duplication
AdLOX	2	7	5	14	1250^a^	1^a^
AiLOX	1	7	5	13	1250^a^	1^a^
CaLOX	2	1	7	10	738^b^	1^b^
CcLOX	3	4	9	16	833^c^	0^c^
GmLOX	5	17	14	36	1100^d^	3^d^
LjLOX	0	3	2	5	315^e^	1^e^
MtLOX	3	18	7	28	375^f^	3^f^

Note: a data were obtained from references 22 and 24; b data were obtained from reference 23; c data were from reference 28; d data were from reference 27; e data were from reference 25; f data were from reference 26.

**Table 2:** GC content of *LOX* genes in seven legumes.

Gene	GC1 content	GC2 content	GC3 content	Average GC content
AdLOX	50.08	38.11	40.28	42.82
AiLOX	49.95	38.26	39.78	42.66
CaLOX	48.44	37.53	31.26	39.08
CcLOX	51.15	38.45	40.69	43.43
GmLOX	50.03	38.48	40.13	43.88
LjLOX	50.81	39.71	42.67	44.4
MtLOX	49.55	37.89	34.61	40.68
