# Supplementary material for: Identification of lipoxygenase (LOX) genes from legumes and their responses in wild type and cultivated peanut upon Aspergillus flavus infection
Source: Sci Rep. 2016 Oct 12;6:35245. doi: 10.1038/srep35245 (PMC5059700; doi:10.1038/srep35245)
Supplement: Supplementary Information [file srep35245-s1.doc]

### Identification of lipoxygenase (LOX) genes from legumes and their responses between wild type and cultivated peanut upon *Aspergillus flavus* infection

### Hui Song^1^, Pengfei Wang^1^, Changsheng Li^1^, Suoyi Han^2^, Javier Lopez-Baltazar ^3^, Xinyou Zhang^2*^, Xingjun Wang^1*^

1 Biotechnology Research Center, Shandong Academy of Agricultural Sciences; Shandong Provincial Key laboratory of Crop Genetic Improvement, Ecology and Physiology, Jinan 250100, PR China

2 Henan Academy of Agricultural Sciences, Zhengzhou 450002, PR China

3 Departamento de Ingenierías, Instituto Tecnológico del Valle de Oaxaca, Oaxaca, Mexico

* Corresponding author: haasz@sohu.com; [xingjunw@hotmail.com](mailto:xingjunw@hotmail.com)

**Table S1** *LOX* genes identified in seven legumes.

| ID | sequence | Chromosomes | Location | Anmio acid length | CDS length | Location of PLAT | Location of Lipoxygenase |
| --- | --- | --- | --- | --- | --- | --- | --- |
| *Arachis duranensis* | |  |  |  |  |  |  |
| Aradu.289WG | partial sequence | 6 | 108886704-108892593 | 623 | 1872 | 90-189 | 202-619 |
| Aradu.951UC | full-length sequence | 9 | 13560051-13567039 | 837 | 2604 | 71-174 | 187-850 |
| Aradu.AC956 | full-length sequence | 8 | 23267762-23276904 | 854 | 2514 | 61-167 | 170-820 |
| Aradu.AE16G | full-length sequence | 9 | 7379421-7386679 | 914 | 2565 | 54-158 | 171-837 |
| Aradu.AS232 | full-length sequence | 10 | 2839109-2844699 | 860 | 2747 | 113-215 | 228-897 |
| Aradu.C3RV0 | full-length sequence | 9 | 33330334-33339787 | 942 | 2583 | 55-163 | 176-843 |
| Aradu.C88Z1 | full-length sequence | 3 | 133033579-133038386 | 865 | 2832 | 129-229 | 241-925 |
| Aradu.FM0YX | full-length sequence | 9 | 13569491-13576130 | 862 | 2598 | 65-170 | 183-848 |
| Aradu.G99LQ | full-length sequence | 6 | 4803755-4810698 | 862 | 2589 | 52-159 | 172-845 |
| Aradu.GJ1CE | partial sequence | 9 | 8510756-8521832 | 803 | 2412 | 58-163 | 176-501/499-781 |
| Aradu.KXZ9V | partial sequence | 8 | 23274432-23281985 | 509 | 1530 | 3-54 | 73-172/169-257/251-508 |
| Aradu.KZX2M | partial sequence | 9 | 111439030-111445210 | 887 | 2664 | 56-163 | 176-367/411-800/797-865 |
| Aradu.Q5K4W | full-length sequence | 3 | 3271850-3290419 | 855 | 4374 | 121-212/1010-1097 | 225-890/1110-1451 |
| Aradu.SK1BS | full-length sequence | 9 | 7355852-7361757 | 867 | 2568 | 56-159 | 172-838 |
| Aradu.TJL9X | full-length sequence | 9 | 13752411-13758989 | 896 | 2604 | 68-172 | 185-850 |
| Aradu.W07KG | full-length sequence | 3 | 119244930-119248414 | 840 | 2691 | 126-224 | 237-879 |
| Aradu.WX5KP | full-length sequence | 8 | 23296802-23300803 | 914 | 2523 | 67-171 | 184-280/262-823 |
| Aradu.XZG8N | full-length sequence | 2 | 68801806-68808005 | 914 | 2745 | 113-215 | 228-897 |
| *Arachis* *ipaënsis* | |  |  |  |  |  |  |
| Araip.2KP3T | partial sequence | 6 | 21107589-21113101 | 578 | 1737 | 124-210 | 223-574 |
| Araip.5F6MD | full-length sequence | 9 | 9136632-9144858 | 882 | 2649 | 56-159 | 172-377/371-865 |
| Araip.849ER | full-length sequence | 2 | 80474641-80481479 | 914 | 2745 | 115-215 | 228-897 |
| Araip.D6PZJ | partial sequence | 9 | 145587000-145594367 | 820 | 2463 | 56-163 | 176-585/582-720/716-783 |
| Araip.DH1Z0 | full-length sequence | 8 | 1480891-1487297 | 855 | 2568 | 61-168 | 181-838 |
| Araip.E99Y9 | partial sequence | 8 | 1487051-1494231 | 863 | 2592 | 67-171 | 184-846 |
| Araip.GV48H | full-length sequence | 9 | 17683757-17690365 | 869 | 2610 | 65-170 | 187-852 |
| Araip.HGI2J | full-length sequence | 6 | 133162764-133172431 | 969 | 2919 | 93-189 | 219-432/430-696/703-952 |
| Araip.J97JQ | full-length sequence | 4 | 125913173-125914171 | 174 | 525 | 4-88 | 101-173 |
| Araip.K56RN | full-length sequence | 9 | 17850863-17858383 | 867 | 2604 | 68-172 | 185-850 |
| Araip.MN7KE | full-length sequence | 3 | 5996999-6016424 | 598 | 1797 | 130-220 | 233-591 |
| Araip.NWR3L | full-length sequence | 3 | 120232772-120237437 | 895 | 2688 | 125-223 | 236-878 |
| Araip.Q7EYZ | full-length sequence | 10 | 4453319-4459459 | 863 | 2592 | 113-215 | 228-834 |
| Araip.Q8LFT | full-length sequence | 9 | 17673837-17681644 | 867 | 2604 | 71-174 | 187-850 |
| Araip.T64GQ | full-length sequence | 9 | 9188959-9197584 | 854 | 2565 | 54-158 | 171-837 |
| Araip.VN0A4 | partial sequence | 9 | 19337891-19344982 | 930 | 2793 | 133-230 | 243-913 |
| Araip.W6TLM | partial sequence | 6 | 13220247-13227169 | 862 | 2589 | 52-159 | 172-845 |
| Araip.X1W86 | full-length sequence | 3 | 134148128-134153671 | 940 | 2826 | 125-227 | 239-923 |
| *Cicer arietinum* |  |  |  |  |  |  |  |
| Ca_02458.1 | truncated domain | Not observed | Not observed | 2298 | 765 | 55-161 | 174-429/426-748 |
| Ca_04554.1 | full-length sequence | Not observed | Not observed | 2577 | 858 | 43-151 | 164-836 |
| Ca_04858.1 | full-length sequence | Not observed | Not observed | 2577 | 858 | 52-157 | 170-841 |
| Ca_07045.1 | full-length sequence | Not observed | Not observed | 2445 | 814 | 119-204 | 217-796 |
| Ca_07906.1 | full-length sequence | Not observed | Not observed | 2799 | 932 | 136-232 | 245-915 |
| Ca_09788.1 | truncated domain | Not observed | Not observed | 2028 | 675 | 68-172 | 185-663 |
| Ca_09789.1 | full-length sequence | Not observed | Not observed | 2127 | 708 | 68-172 | 185-687 |
| Ca_09789.2 | alternative splicing | Not observed | Not observed | 1602 | 533 | 68-172 | 185-533 |
| Ca_12521.1 | truncated domain | Not observed | Not observed | 1359 | 452 | 71-173 | 186-437 |
| Ca_16484.1 | full-length sequence | Not observed | Not observed | 2724 | 907 | 122-207 | 220-890 |
| Ca_16484.2 | alternative splicing | Not observed | Not observed | 2718 | 905 | 123-205 | 218-888 |
| Ca_17333.1 | full-length sequence | Not observed | Not observed | 2487 | 828 | 44-139 | 151-811 |
| Ca_17334.1 | full-length sequence | Not observed | Not observed | 2703 | 900 | 107-202 | 214-883 |
| Ca_17335.1 | full-length sequence | Not observed | Not observed | 2574 | 867 | 62-160 | 173-840 |
| Ca_18748.1 | truncated domain | Not observed | Not observed | 1221 | 406 | 18-115 | 119-161/153-327/325-389 |
| Ca_18875.1 | full-length sequence | Not observed | Not observed | 2328 | 775 | 53-143 | 155-775 |
| Ca_19085.1 | truncated domain | Not observed | Not observed | 651 | 216 | 66-170 | 168-213 |
| *Cajanus cajan* |  |  |  |  |  |  |  |
| C.cajan_06814 | full-length sequence | Not observed | Not observed | 2583 | 860 | 52-160 | 173-844 |
| C.cajan_07530 | full-length sequence | Not observed | Not observed | 2583 | 860 | 49-156 | 169-839 |
| C.cajan_10184 | full-length sequence | Not observed | Not observed | 2577 | 858 | 55-161 | 174-842 |
| C.cajan_11005 | full-length sequence | Not observed | Not observed | 2709 | 902 | 117-211 | 220-886 |
| C.cajan_13398 | partial sequence | Not observed | Not observed | 6684 | 2227 | 598-701/1429-1532 | 1-524/714-1213/1209-1329/1545-2210 |
| C.cajan_13399 | full-length sequence | Not observed | Not observed | 2586 | 861 | 66-170 | 183-845 |
| C.cajan_17695 | truncated domain | Not observed | Not observed | 1764 | 587 | 3-54 | 67-531/529-571 |
| C.cajan_17696 | full-length sequence | Not observed | Not observed | 2595 | 864 | 67-171 | 184-848 |
| C.cajan_17697 | full-length sequence | Not observed | Not observed | 2592 | 863 | 64-168 | 181-847 |
| C.cajan_17698 | truncated domain | Not observed | Not observed | 1611 | 536 | 70-164 | 144-450/447-520 |
| C.cajan_17700 | full-length sequence | Not observed | Not observed | 2592 | 863 | 69-171 | 184-847 |
| C.cajan_19929 | full-length sequence | Not observed | Not observed | 2496 | 831 | 63-151 | 164-815 |
| C.cajan_20034 | full-length sequence | Not observed | Not observed | 2559 | 852 | 66-152 | 165-836 |
| C.cajan_21236 | full-length sequence | Not observed | Not observed | 2706 | 901 | 116-205 | 218-885 |
| C.cajan_21238 | full-length sequence | Not observed | Not observed | 2712 | 903 | 120-205 | 218-887 |
| C.cajan_23743 | truncated domain | Not observed | Not observed | 1890 | 629 | 60-165 | 178-615 |
| C.cajan_24301 | full-length sequence | Not observed | Not observed | 2733 | 910 | 124-211 | 224-894 |
| C.cajan_27693 | full-length sequence | Not observed | Not observed | 2766 | 921 | 128-225 | 238-905 |
| C.cajan_42248 | full-length sequence | Not observed | Not observed | 2547 | 848 | 48-146 | 159-832 |
| C.cajan_44133 | full-length sequence | Not observed | Not observed | 2715 | 904 | 119-209 | 222-888 |
| *Glycine max* |  |  |  |  |  |  |  |
| Glyma.03G091000.1 | full-length sequence | 3 | 27013300-27020898 | 2601 | 866 | 60-164 | 177-843 |
| Glyma.03G237300.1 | full-length sequence | 3 | 43723483-43730370 | 2577 | 858 | 49-157 | 170-841 |
| Glyma.03G237300.2 | alternative splicing | 3 | 43723483-43730370 | 2457 | 818 | 49-157 | 170-245/239-801 |
| Glyma.03G264300.1 | full-length sequence | 3 | 45704553-45709164 | 2706 | 901 | 117-211 | 221-884 |
| Glyma.07G006700.1 | truncated domain | 7 | 491733-496064 | 1713 | 570 | 66-171 | 184-569 |
| Glyma.07G006900.1 | full-length sequence | 7 | 503752-509348 | 2580 | 859 | 68-172 | 185-842 |
| Glyma.07G007000.1 | full-length sequence | 7 | 509819-514700 | 2595 | 864 | 66-170 | 183-847 |
| Glyma.07G007000.2 | alternative splicing | 7 | 509819-514700 | 1854 | 617 | 66-170 | 183-617 |
| Glyma.07G007100.1 | truncated domain | 7 | 529398-533680 | 1368 | 455 | 18-110 | 123-345/344-455 |
| Glyma.07G034800.1 | full-length sequence | 7 | 2763836-2770113 | 2598 | 865 | 69-172 | 185-848 |
| Glyma.07G034800.2 | alternative splicing | 7 | 2763836-2770113 | 1374 | 457 | 68-172 | 185-452 |
| Glyma.07G034800.3 | alternative splicing | 7 | 2763836-2770113 | 1848 | 615 | 68-172 | 185-615 |
| Glyma.07G034900.1 | full-length sequence | 7 | 2782231-2787974 | 2607 | 868 | 69-173 | 186-851 |
| Glyma.07G039900.1 | full-length sequence | 7 | 3288274-3294751 | 2784 | 927 | 130-228 | 241-910 |
| Glyma.07G196800.1 | full-length sequence | 7 | 36509005-36518982 | 2733 | 910 | 135-231 | 244-893 |
| Glyma.08G102900.1 | full-length sequence | 8 | 7886865-7892029 | 2766 | 921 | 127-221 | 234-904 |
| Glyma.08G189200.1 | full-length sequence | 8 | 15172904-15178499 | 2583 | 860 | 61-165 | 178-843 |
| Glyma.08G189200.2 | alternative splicing | 8 | 15172904-15178499 | 2478 | 825 | 61-165 | 178-543/540-808 |
| Glyma.08G189300.1 | full-length sequence | 8 | 15186020-15191205 | 2574 | 857 | 50-157 | 171-840 |
| Glyma.08G189400.1 | full-length sequence | 8 | 15193038-15201472 | 2541 | 846 | 61-166 | 179-829 |
| Glyma.08G189500.1 | full-length sequence | 8 | 15206364-15212242 | 2604 | 867 | 66-171 | 184-850 |
| Glyma.08G189600.1 | full-length sequence | 8 | 15235197-15239971 | 2601 | 866 | 68-172 | 185-849 |
| Glyma.08G189800.1 | full-length sequence | 8 | 15249052-15254212 | 2607 | 868 | 70-172 | 185-851 |
| Glyma.10G153900.1 | full-length sequence | 10 | 38898358-38904608 | 2598 | 865 | 56-164 | 177-848 |
| Glyma.10G153900.2 | alternative splicing | 10 | 38898358-38904608 | 1848 | 615 | 56-164 | 177-615 |
| Glyma.11G130200.1 | full-length sequence | 11 | 9903923-9913136 | 2721 | 906 | 121-206 | 219-889 |
| Glyma.11G130300.1 | full-length sequence | 11 | 9925260-9936297 | 2706 | 901 | 113-207 | 220-884 |
| Glyma.12G054700.1 | full-length sequence | 12 | 3949453-3957386 | 2745 | 914 | 129-214 | 227-897 |
| Glyma.13G030300.1 | full-length sequence | 13 | 9773782-9780355 | 2757 | 918 | 124-216 | 229-901 |
| Glyma.13G030300.2 | alternative splicing | 13 | 9773782-9780355 | 2115 | 704 | Not observed | 15-687 |
| Glyma.13G075900.1 | full-length sequence | 13 | 17965521-17975820 | 2736 | 911 | 120-213 | 226-894 |
| Glyma.13G075900.2 | alternative splicing | 13 | 17965521-17975820 | 2412 | 803 | 120-213 | 226-295/292-786 |
| Glyma.13G347500.1 | full-length sequence | 13 | 43761727-43766023 | 2601 | 866 | 72-174 | 187-849 |
| Glyma.13G347600.1 | full-length sequence | 13 | 43769116-43772944 | 2481 | 826 | 40-144 | 157-809 |
| Glyma.13G347600.2 | alternative splicing | 13 | 43769116-43772944 | 2109 | 702 | 40-144 | 157-681 |
| Glyma.13G347700.1 | full-length sequence | 13 | 43773475-43780320 | 2562 | 853 | 56-158 | 171-836 |
| Glyma.13G347800.1 | full-length sequence | 13 | 43797692-43803266 | 2469 | 822 | 58-161 | 174-821 |
| Glyma.15G026300.1 | full-length sequence | 15 | 2123754-2128104 | 2574 | 857 | 58-162 | 175-840 |
| Glyma.15G026400.1 | full-length sequence | 15 | 2130531-2134563 | 2571 | 856 | 58-161 | 174-839 |
| Glyma.15G026500.1 | full-length sequence | 15 | 2142191-2147489 | 2562 | 853 | 56-158 | 171-836 |
| Glyma.16G008700.1 | full-length sequence | 16 | 735622-742337 | 2769 | 922 | 125-223 | 236-905 |
| Glyma.16G082600.1 | full-length sequence | 16 | 9227831-9237983 | 2586 | 861 | 56-160 | 173-839 |
| Glyma.19G263300.1 | full-length sequence | 19 | 50591809-50596423 | 2700 | 899 | 115-208 | 218-882 |
| Glyma.20G053700.1 | full-length sequence | 20 | 12341598-12348808 | 2580 | 859 | 64-160 | 173-842 |
| Glyma.20G053700.2 | alternative splicing | 20 | 12341598-12348808 | 1824 | 607 | 64-160 | 173-607 |
| Glyma.20G054000.1 | full-length sequence | 20 | 12411805-12423973 | 2712 | 903 | 104-200 | 213-886 |
| Glyma.20G054000.2 | alternative splicing | 20 | 12411805-12423973 | 2688 | 895 | 104-200 | 213-878 |
| Glyma.20G054100.1 | full-length sequence | 20 | 12486755-12493110 | 2583 | 860 | 65-161 | 174-843 |
| Glyma.20G144600.1 | full-length sequence | 20 | 38316738-38324822 | 2577 | 858 | 48-153 | 166-836 |
| Glyma.20G144600.2 | alternative splicing | 20 | 38316738-38324822 | 2283 | 760 | 2-55 | 68-738 |
| Glyma.20G144600.3 | alternative splicing | 20 | 38316738-38324822 | 2577 | 858 | 48-153 | 166-836 |
| Glyma.U039300.1 | partial sequence | scaffold_442 | 4942-10906 | 1368 | 456 | 40-145 | 158-411 |
| Glyma.U039300.2 | alternative splicing | scaffold_442 | 4942-10906 | 1365 | 455 | 40-145 | 158-411 |
| Glyma.U039300.3 | alternative splicing | scaffold_442 | 4942-10906 | 1365 | 455 | 40-145 | 158-411 |
| *Lotus japonicus* |  |  |  |  |  |  |  |
| chr1.CM0544.250.r2.m | partial sequence | Not observed | Not observed | 1805 | 601 | 48-154 | 167-601 |
| chr3.CM0106.830.r2.d | partial sequence | Not observed | Not observed | 1492 | 497 | 46-149 | 162-492 |
| chr3.CM0106.990.r2.d | truncated domain | Not observed | Not observed | 2583 | 860 | 61-165 | 178-843 |
| chr3.CM0115.240.r2.m | full-length sequence | Not observed | Not observed | 2586 | 861 | 69-173 | 186-844 |
| chr3.CM0115.260.r2.m | full-length sequence | Not observed | Not observed | 2532 | 843 | 39-145 | 158-826 |
| chr3.CM0115.330.r2.m | full-length sequence | Not observed | Not observed | 2613 | 870 | 70-174 | 187-853 |
| chr3.CM0115.370.r2.m | partial sequence | Not observed | Not observed | 2382 | 794 | 1-98 | 111-777 |
| chr3.CM0416.980.r2.m | full-length sequence | Not observed | Not observed | 2703 | 900 | 104-201 | 214-883 |
| chr3.CM0634.600.r2.m | partial sequence | Not observed | Not observed | 453 | 151 | Not observed | 1-133 |
| chr6.LjT22E11.10.r2.m | partial sequence | Not observed | Not observed | 1711 | 570 | 47-140 | 153-570 |
| LjB17J04.70.r2.m | full-length sequence | Not observed | Not observed | 2814 | 937 | 141-238 | 251-920 |
| *Medicago truncatila* | |  |  |  |  |  |  |
| Medtr0248s0010.1 | truncated domain | scaffold0248 | 12391-12918 | 258 | 85 |  |  |
| Medtr1g083020.1 | full-length sequence | 1 | 36920655-36927114 | 2577 | 858 | 52-157 | 170-841 |
| Medtr1g104650.1 | full-length sequence | 1 | 47126128-47132503 | 2571 | 856 | 42-150 | 163-834 |
| Medtr1g104650.2 | alternative splicing | 1 | 47126128-47132437 | 2571 | 856 | 42-150 | 163-834 |
| Medtr1g104650.3 | alternative splicing | 1 | 47126095-47132583 | 2571 | 856 | 42-150 | 163-834 |
| Medtr2g099560.1 | full-length sequence | 2 | 42686173-42690338 | 2592 | 863 | 66-170 | 183-846 |
| Medtr2g099570.1 | full-length sequence | 2 | 42691747-42697437 | 2586 | 861 | 61-165 | 178-844 |
| Medtr3g079420.1 | full-length sequence | 3 | 35705582-35709331 | 2598 | 865 | 67-164 | 177-848 |
| Medtr3g079450.1 | full-length sequence | 3 | 35715739-35723794 | 2709 | 902 | 107-202 | 215-885 |
| Medtr3g079450.2 | alternative splicing | 3 | 35719843-35725294 | 1953 | 650 | 106-202 | 215-650 |
| Medtr3g081130.1 | full-length sequence | 3 | 36742900-36746629 | 2577 | 858 | 66-164 | 177-841 |
| Medtr3g081130.2 | alternative splicing | 3 | 36742841-36746629 | 2361 | 786 | 2-92 | 105-769 |
| Medtr3g479460.1 | full-length sequence | 3 | 35725453-35729835 | 2712 | 903 | 107-203 | 216-886 |
| Medtr4g066170.1 | full-length sequence | 4 | 24959921-24968304 | 2721 | 906 | 120-208 | 221-889 |
| Medtr4g088810.1 | full-length sequence | 4 | 35409075-35415156 | 2688 | 895 | 118-214 | 227-878 |
| Medtr5g024020.1 | full-length sequence | 5 | 9600482-9607033 | 2604 | 867 | 68-173 | 186-850 |
| Medtr5g024020.2 | alternative splicing | 5 | 36742841-36746629 | 2496 | 831 | 68-173 | 186-394/390-814 |
| Medtr7g113410.1 | full-length sequence | 7 | 46706228-46714602 | 2604 | 867 | 57-165 | 178-850 |
| Medtr8g018420.1 | full-length sequence | 8 | 6261408-6266171 | 2571 | 856 | 58-160 | 173-839 |
| Medtr8g018430.1 | full-length sequence | 8 | 6273027-6277365 | 2586 | 861 | 64-165 | 178-844 |
| Medtr8g018450.1 | full-length sequence | 8 | 6287218-6291683 | 2487 | 828 | 63-158 | 171-311/306-811 |
| Medtr8g018510.1 | full-length sequence | 8 | 6319619-6324603 | 2610 | 869 | 71-173 | 186-852 |
| Medtr8g018520.1 | full-length sequence | 8 | 6330368-6336843 | 2607 | 868 | 72-172 | 185-851 |
| Medtr8g018550.1 | full-length sequence | 8 | 6350475-6356214 | 2607 | 868 | 70-172 | 185-851 |
| Medtr8g018570.1 | full-length sequence | 8 | 6361499-6365542 | 2613 | 870 | 74-174 | 187-853 |
| Medtr8g018590.1 | full-length sequence | 8 | 6368207-6373263 | 2472 | 823 | 41-141 | 154-259/249-806 |
| Medtr8g018620.1 | full-length sequence | 8 | 6387548-6393103 | 2424 | 807 | 51-154 | 167-309/304-790 |
| Medtr8g018650.1 | full-length sequence | 8 | 6408474-6412307 | 2424 | 807 | 51-154 | 167-309/304-790 |
| Medtr8g018690.1 | full-length sequence | 8 | 6422466-6426852 | 2598 | 865 | 71-174 | 187-848 |
| Medtr8g018690.2 | alternative splicing | 8 | 6422487-6426852 | 2538 | 845 | 71-174 | 174-828 |
| Medtr8g018730.1 | full-length sequence | 8 | 6448981-6454397 | 2595 | 864 | 66-170 | 183-847 |
| Medtr8g018730.2 | alternative splicing | 8 | 6448992-6454307 | 2505 | 834 | 66-170 | 183-281/276-817 |
| Medtr8g018735.1 | full-length sequence | 8 | 6455504-6459438 | 2598 | 865 | 67-171 | 184-848 |
| Medtr8g018735.2 | alternative splicing | 8 | 6455504-6459438 | 1929 | 642 | 67-171 | 184-620 |
| Medtr8g020970.1 | full-length sequence | 8 | 7436326-7443707 | 2784 | 927 | 128-226 | 239-910 |
| Medtr8g020970.2 | alternative splicing | 8 | 7437598-7443568 | 2031 | 676 | 128-226 | 239-676 |
| Medtr8g020990.1 | full-length sequence | 8 | 7457373-7463228 | 2607 | 868 | 68-172 | 185-851 |
| Medtr8g080230.1 | full-length sequence | 8 | 34466671-34473858 | 2751 | 916 | 115-214 | 227-899 |

**Table S2** Codon usage in seven legumes.

|  |  | AdLOX | | AiLOX | | CaLOX | | CcLOX | | GmLOX | | LjLOX | | MtLOX | |
| --- | --- | --- | --- | --- | --- | --- | --- | --- | --- | --- | --- | --- | --- | --- | --- |
| Amino acid | Codon | Number of codon | RSCU | Number of codon | RSCU | Number of codon | RSCU | Number of codon | RSCU | Number of codon | RSCU | Number of codon | RSCU | Number of codon | RSCU |
| Phe | UUU | 266 | **1.07** | 244 | **1.01** | 229 | **1.26** | 311 | **1.05** | 701 | **1.05** | 79 | 0.92 | 613 | **1.19** |
|  | UUC | 231 | 0.93 | 238 | 0.99 | 134 | 0.74 | 282 | 0.95 | 636 | 0.95 | 92 | **1.08** | 421 | 0.81 |
| Leu | UUA | 157 | 0.9 | 118 | 0.64 | 156 | **1.1** | 166 | 0.71 | 401 | 0.77 | 55 | 0.76 | 347 | 0.88 |
|  | UUG | 228 | **1.31** | 309 | **1.68** | 216 | **1.52** | 326 | **1.4** | 808 | **1.56** | 129 | **1.77** | 658 | **1.68** |
|  | CUU | 291 | **1.67** | 291 | **1.58** | 238 | **1.68** | 390 | **1.67** | 859 | **1.66** | 114 | **1.57** | 694 | **1.77** |
|  | CUC | 156 | 0.89 | 152 | 0.83 | 97 | 0.68 | 223 | 0.95 | 442 | 0.85 | 54 | 0.74 | 260 | 0.66 |
|  | CUA | 134 | 0.77 | 136 | 0.74 | 101 | 0.71 | 158 | 0.68 | 340 | 0.66 | 41 | 0.56 | 260 | 0.66 |
|  | CUG | 81 | 0.46 | 98 | 0.53 | 43 | 0.3 | 139 | 0.59 | 259 | 0.5 | 44 | 0.6 | 134 | 0.34 |
| Ile | AUU | 318 | **1.41** | 361 | **1.43** | 284 | **1.47** | 385 | **1.3** | 901 | **1.31** | 131 | **1.4** | 732 | **1.38** |
|  | AUC | 201 | 0.89 | 198 | 0.78 | 106 | 0.55 | 241 | 0.81 | 596 | 0.86 | 92 | 0.98 | 394 | 0.75 |
|  | AUA | 159 | 0.7 | 199 | 0.79 | 188 | 0.98 | 263 | 0.89 | 573 | 0.83 | 58 | 0.62 | 460 | 0.87 |
| Met | AUG | 155 | 1 | 207 | 1 | 126 | 1 | 222 | 1 | 520 | 1 | 61 | 1 | 373 | 1 |
| Val | GUU | 255 | **1.78** | 304 | **1.74** | 203 | **1.8** | 302 | **1.46** | 710 | **1.56** | 107 | **1.55** | 650 | **1.78** |
|  | GUC | 104 | 0.73 | 110 | 0.63 | 72 | 0.64 | 148 | 0.71 | 316 | 0.69 | 50 | 0.72 | 223 | 0.61 |
|  | GUA | 84 | 0.59 | 86 | 0.49 | 66 | 0.59 | 120 | 0.58 | 217 | 0.48 | 26 | 0.38 | 218 | 0.6 |
|  | GUG | 130 | 0.91 | 199 | **1.14** | 110 | 0.98 | 259 | **1.25** | 583 | **1.28** | 93 | **1.35** | 367 | **1.01** |
| Ser | UCU | 214 | **1.15** | 189 | **1.39** | 140 | **1.38** | 227 | **1.39** | 531 | **1.44** | 95 | **1.69** | 398 | **1.4** |
|  | UCC | 208 | **1.12** | 102 | 0.75 | 48 | 0.47 | 101 | 0.62 | 236 | 0.64 | 41 | 0.73 | 155 | 0.54 |
|  | UCA | 216 | **1.16** | 160 | **1.18** | 177 | **1.74** | 226 | **1.38** | 502 | **1.36** | 56 | 0.99 | 392 | **1.38** |
|  | UCG | 70 | 0.38 | 40 | 0.29 | 25 | 0.25 | 43 | 0.26 | 101 | 0.27 | 9 | 0.16 | 72 | 0.25 |
| Pro | CCU | 212 | **1.35** | 268 | **1.57** | 204 | **1.61** | 324 | **1.48** | 709 | **1.49** | 121 | **1.7** | 542 | **1.54** |
|  | CCC | 123 | 0.78 | 88 | 0.51 | 28 | 0.22 | 114 | 0.52 | 222 | 0.47 | 35 | 0.49 | 98 | 0.28 |
|  | CCA | 238 | **1.51** | 272 | **1.59** | 258 | **2.03** | 381 | **1.74** | 861 | **1.81** | 109 | **1.54** | 683 | **1.94** |
|  | CCG | 56 | 0.36 | 56 | 0.33 | 18 | 0.14 | 57 | 0.26 | 112 | 0.24 | 19 | 0.27 | 86 | 0.24 |
| Thr | ACU | 245 | **1.54** | 249 | **1.54** | 185 | **1.45** | 232 | **1.21** | 602 | **1.35** | 86 | **1.54** | 546 | **1.53** |
|  | ACC | 134 | 0.84 | 129 | 0.8 | 78 | 0.61 | 166 | 0.86 | 411 | 0.92 | 60 | **1.08** | 273 | 0.76 |
|  | ACA | 219 | **1.38** | 237 | **1.47** | 227 | **1.78** | 296 | **1.54** | 670 | **1.5** | 67 | **1.2** | 520 | **1.46** |
|  | ACG | 38 | 0.24 | 32 | 0.2 | 19 | 0.15 | 76 | 0.39 | 102 | 0.23 | 10 | 0.18 | 90 | 0.25 |
| Ala | GCU | 229 | **1.44** | 252 | **1.5** | 196 | **1.77** | 303 | **1.46** | 702 | **1.53** | 128 | **1.68** | 670 | **1.86** |
|  | GCC | 163 | **1.03** | 126 | 0.75 | 52 | 0.47 | 162 | 0.78 | 361 | 0.79 | 65 | 0.86 | 201 | 0.56 |
|  | GCA | 207 | **1.3** | 251 | **1.49** | 185 | **1.67** | 297 | **1.43** | 690 | **1.5** | 96 | **1.26** | 506 | **1.41** |
|  | GCG | 36 | 0.23 | 44 | 0.26 | 11 | 0.1 | 68 | 0.33 | 84 | 0.18 | 15 | 0.2 | 62 | 0.17 |
| Tyr | UAU | 255 | **1.15** | 258 | **1.1** | 221 | **1.39** | 314 | **1.19** | 719 | **1.19** | 97 | **1.05** | 588 | **1.17** |
|  | UAC | 188 | 0.85 | 213 | 0.9 | 98 | 0.61 | 213 | 0.81 | 486 | 0.81 | 88 | 0.95 | 418 | 0.83 |
| His | CAU | 292 | **1.25** | 190 | **1.27** | 183 | **1.48** | 259 | **1.26** | 576 | **1.29** | 66 | **1.31** | 448 | **1.38** |
|  | CAC | 177 | 0.75 | 110 | 0.73 | 65 | 0.52 | 153 | 0.74 | 315 | 0.71 | 35 | 0.69 | 199 | 0.62 |
| Gln | CAA | 357 | **1.42** | 255 | **1.4** | 216 | **1.59** | 317 | **1.44** | 692 | **1.42** | 70 | **1.08** | 609 | **1.62** |
|  | CAG | 145 | 0.58 | 110 | 0.6 | 56 | 0.41 | 124 | 0.56 | 284 | 0.58 | 60 | 0.92 | 141 | 0.38 |
| Asn | AAU | 314 | **1.08** | 329 | **1.14** | 272 | **1.41** | 365 | **1.14** | 856 | **1.17** | 119 | **1.09** | 769 | **1.29** |
|  | AAC | 267 | 0.92 | 246 | 0.86 | 114 | 0.59 | 274 | 0.86 | 611 | 0.83 | 100 | 0.91 | 420 | 0.71 |
| Lys | AAA | 338 | 1 | 325 | 0.95 | 339 | **1.17** | 419 | 0.94 | 932 | 0.9 | 116 | 0.87 | 811 | **1.03** |
|  | AAG | 341 | 1 | 360 | **1.05** | 239 | 0.83 | 475 | **1.06** | 1131 | 1.1 | 150 | **1.13** | 769 | 0.97 |
| Asp | GAU | 354 | **1.3** | 462 | **1.36** | 330 | **1.36** | 522 | **1.3** | 1198 | **1.32** | 181 | **1.37** | 1051 | **1.43** |
|  | GAC | 191 | 0.7 | 219 | 0.64 | 156 | 0.64 | 282 | 0.7 | 621 | 0.68 | 83 | 0.63 | 414 | 0.57 |
| Glu | GAA | 391 | **1.16** | 397 | **1.07** | 375 | **1.23** | 485 | 1 | 1064 | **1.02** | 119 | 0.89 | 932 | **1.15** |
|  | GAG | 284 | 0.84 | 346 | 0.93 | 234 | 0.77 | 486 | 1 | 1028 | 0.98 | 148 | **1.11** | 687 | 0.85 |
| Cys | UGU | 193 | **1.01** | 39 | 0.85 | 43 | **1.28** | 48 | 0.9 | 117 | 0.93 | 7 | 0.48 | 67 | 0.86 |
|  | UGC | 189 | 0.99 | 53 | **1.15** | 24 | 0.72 | 59 | **1.1** | 135 | **1.07** | 22 | **1.52** | 88 | **1.14** |
| Trp | UGG | 301 | 1 | 182 | 1 | 159 | 1 | 255 | 1 | 573 | 1 | 72 | 1 | 420 | 1 |
| Arg | CGU | 74 | 0.52 | 61 | 0.65 | 60 | 0.89 | 96 | 0.8 | 187 | 0.72 | 29 | 0.75 | 146 | 0.77 |
|  | CGC | 65 | 0.46 | 57 | 0.61 | 20 | 0.3 | 67 | 0.56 | 162 | 0.62 | 34 | 0.88 | 97 | 0.51 |
|  | CGA | 94 | 0.66 | 32 | 0.34 | 33 | 0.49 | 63 | 0.53 | 125 | 0.48 | 19 | 0.49 | 120 | 0.63 |
|  | CGG | 46 | 0.32 | 26 | 0.28 | 11 | 0.16 | 24 | 0.2 | 53 | 0.2 | 12 | 0.31 | 33 | 0.17 |
| Ser | AGU | 224 | **1.21** | 189 | **1.39** | 141 | **1.39** | 223 | **1.36** | 473 | **1.28** | 66 | **1.17** | 440 | **1.55** |
|  | AGC | 182 | 0.98 | 134 | 0.99 | 78 | 0.77 | 161 | 0.98 | 370 | 1 | 71 | **1.26** | 251 | 0.88 |
| Arg | AGA | 365 | **2.56** | 246 | **2.61** | 186 | **2.77** | 260 | **2.17** | 592 | **2.27** | 80 | **2.07** | 478 | **2.52** |
|  | AGG | 212 | **1.49** | 143 | **1.52** | 93 | **1.38** | 208 | **1.74** | 443 | **1.7** | 58 | **1.5** | 264 | **1.39** |
| Gly | GGU | 201 | **1.01** | 217 | **1.18** | 165 | **1.4** | 234 | **1.09** | 601 | **1.19** | 82 | **1.21** | 490 | **1.3** |
|  | GGC | 132 | 0.67 | 119 | 0.65 | 60 | 0.51 | 145 | 0.68 | 314 | 0.62 | 39 | 0.58 | 172 | 0.46 |
|  | GGA | 335 | **1.69** | 275 | **1.5** | 199 | **1.69** | 312 | **1.46** | 746 | **1.48** | 107 | **1.58** | 688 | **1.83** |
|  | GGG | 125 | 0.63 | 123 | 0.67 | 47 | 0.4 | 166 | 0.77 | 351 | 0.7 | 43 | 0.63 | 155 | 0.41 |

Note: Body indicates that the codon is more frequently used than expected.

**Table S3** qRT-PCR primers used in this study.

| ID | Primers | Tm | Length |
| --- | --- | --- | --- |
| Aradu.AE16G | GCCATTCGTGATAGCAAGTAACAG | 66.1 | 258 |
|  | TCCGCAATCGCCATTCCT | 66.1 |  |
| Aradu.C3RV0 | CAACTCATAAGTGCCGTCAA | 62.3 | 169 |
|  | CTGGTGTTCCTATCTCCTCAT | 62.6 |  |
| Aradu.C88Z1 | CAGGGTCAGAGTCAGAGTCA | 62 | 205 |
|  | ACACCGTTGATGAAGTATTCC | 62.2 |  |
| Aradu.KXZ9V | AGACCTTAACTCTTGAAGATGTT | 61.4 | 129 |
|  | GCACCCCAGCATTTATCT | 61.3 |  |
| Aradu.KZX2M | ACTTGTTAGGAATGATGGTGAGAG | 64.3 | 234 |
|  | CTGGAGACGACGAATGATGAC | 64.6 |  |

**
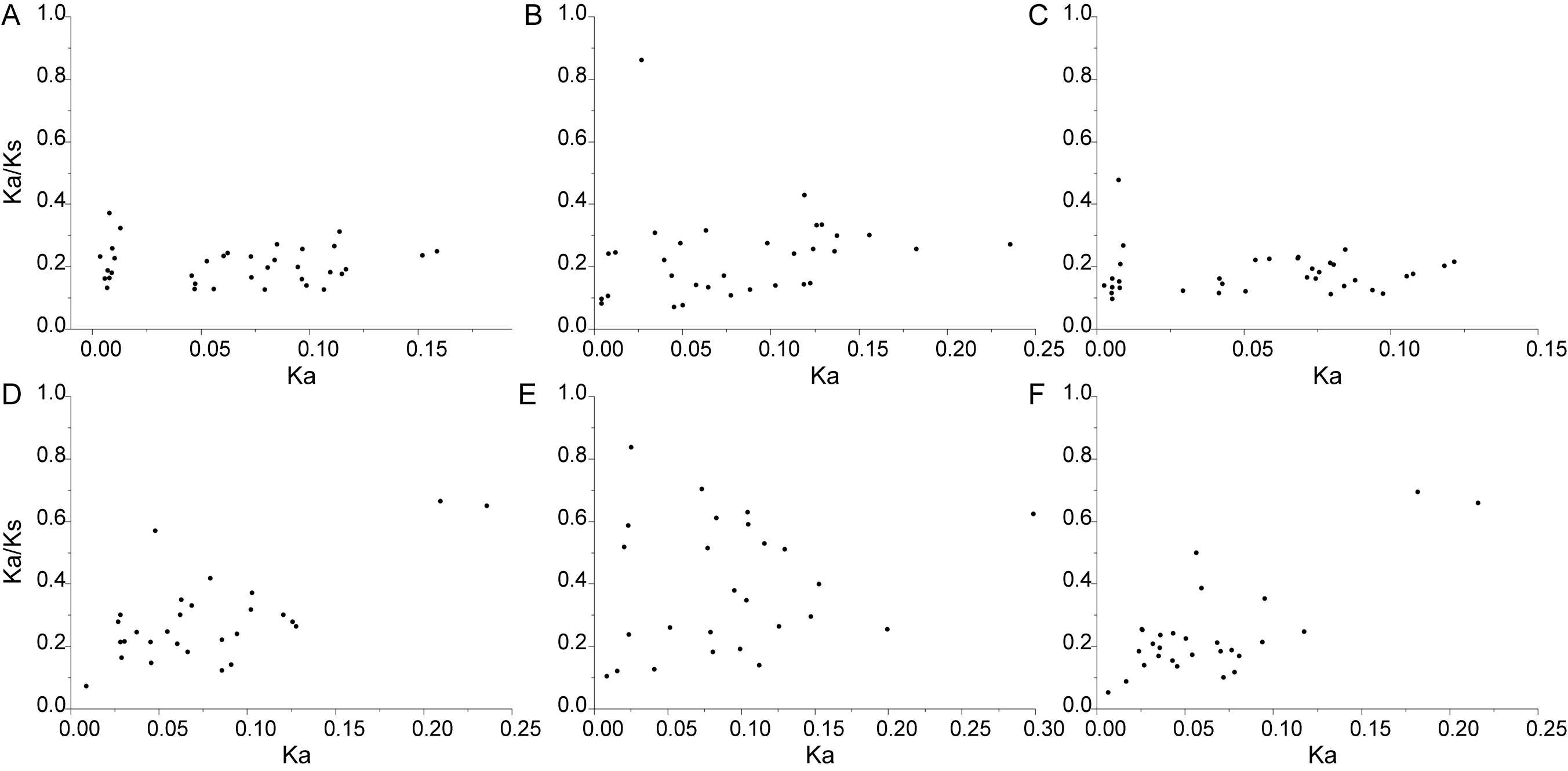
**

**Figure S1** Comparison of *K*_a_/*K*_s_ values among full-length, [polycystin-1](https://en.wikipedia.org/wiki/Polycystin-1), [lipoxygenase](https://en.wikipedia.org/wiki/Lipoxygenase), [alpha-toxin](https://en.wikipedia.org/wiki/Clostridium_perfringens_alpha_toxin) (PLAT) and lipoxygenase domains.

A: *A. duranensis* *LOX* full-length gene; B: *A. duranensis* PLAT domain; C: *A. duranensis* lipoxygenase domain; D: *A.* *ipaënsis* *LOX* full-length gene; E: *A.* *ipaënsis* PLAT domain; F: *A.* *ipaënsis* lipoxygenase domain.


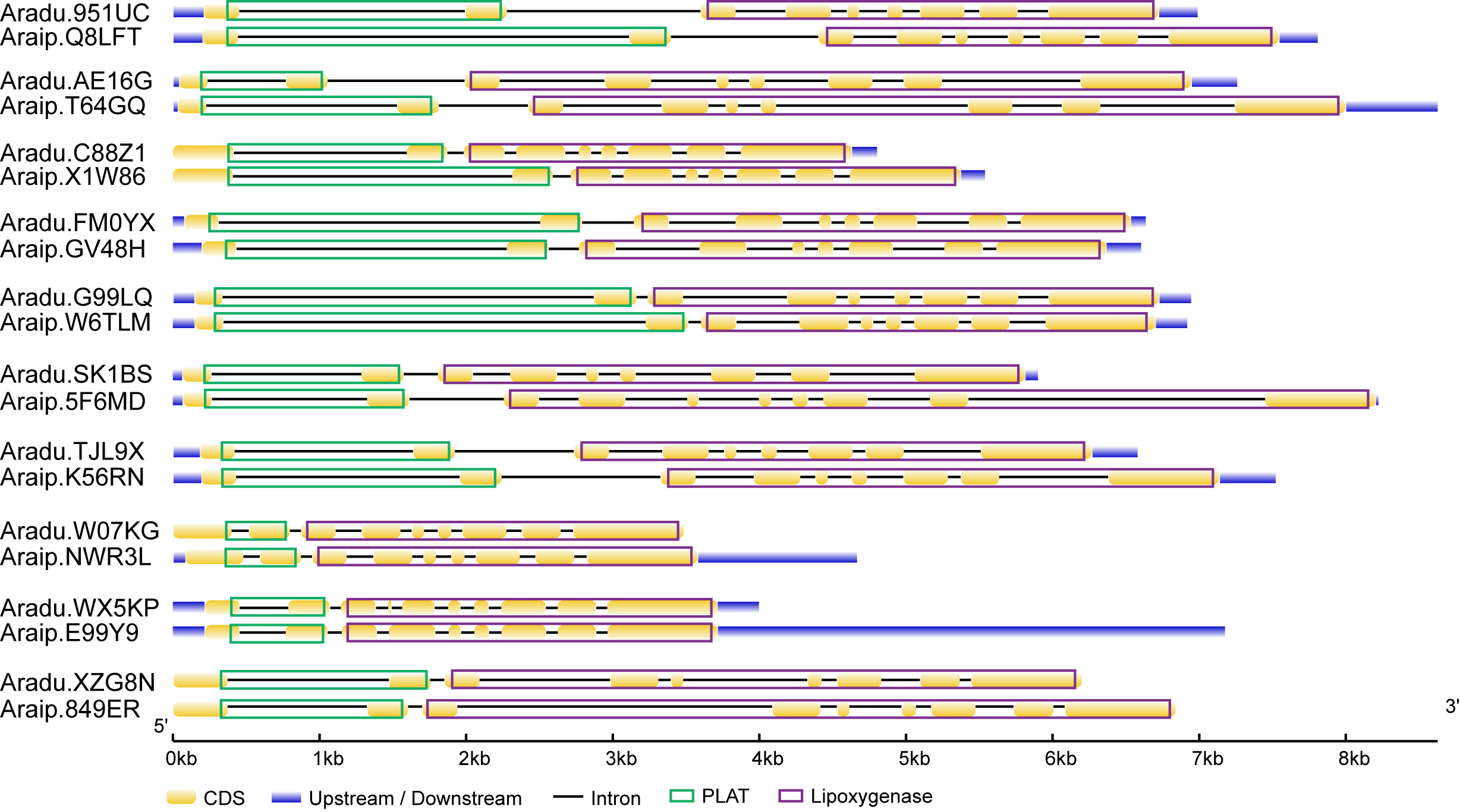


**Figure S2** Gene structure of homologous *LOX* genes in *A. duranensis* and *A.* *ipaënsis*.

Yellow bar indicates CDS sequences, blue bar indicates upstream or downstream sequences, black bar indicates intron sequences, green box indicates PLAT coding sequences, purple box indicates Lipoxygenase sequences.

**
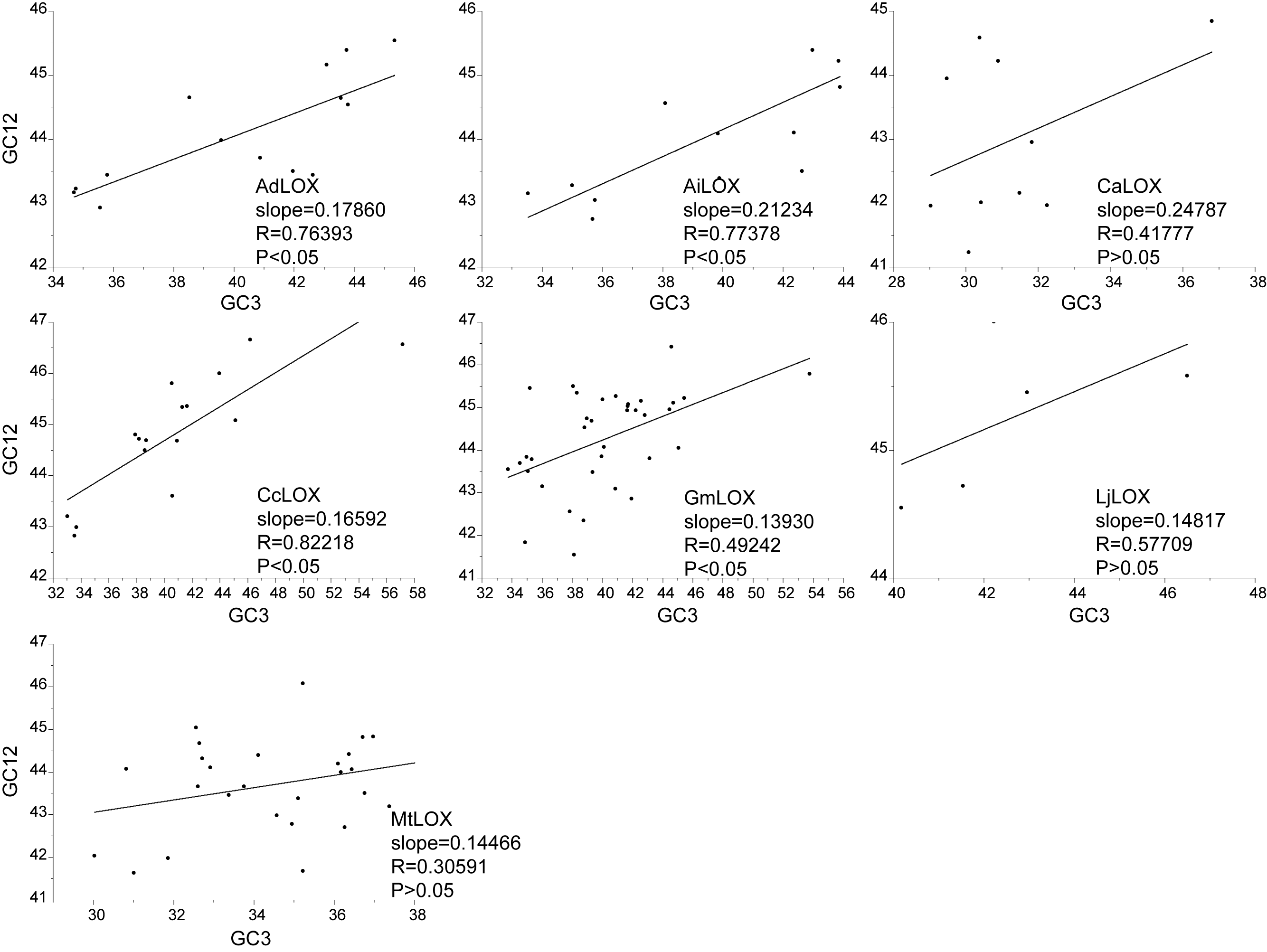
**

**Figure S3** Neutrality plot analyses.

**
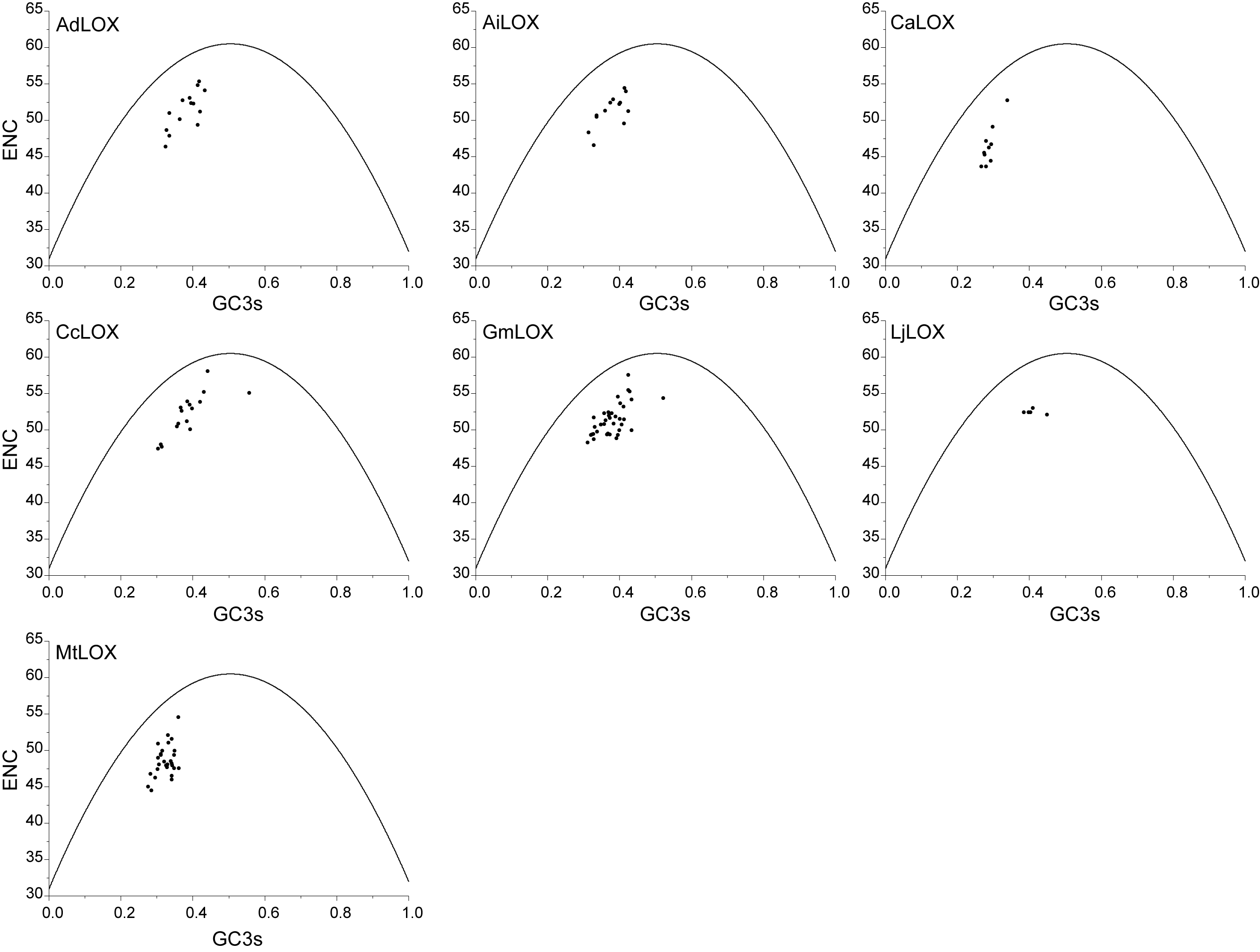
**

**Figure S4** Relationship between GC3s and ENC.
